# Supplementary material for: Causal Relationship between Aging and Anorexia Nervosa: A White-Matter-Microstructure-Mediated Mendelian Randomization Analysis
Source: Biomedicines. 2024 Aug 16;12(8):1874. doi: 10.3390/biomedicines12081874 (PMC11351342; doi:10.3390/biomedicines12081874)
Supplement: Supplementary file 1 [file biomedicines-12-01874-s001.zip › Supplementary Figure/Figure S4/Singe SNP plot of MR analysis for TL to ubm-b-1982.pdf]

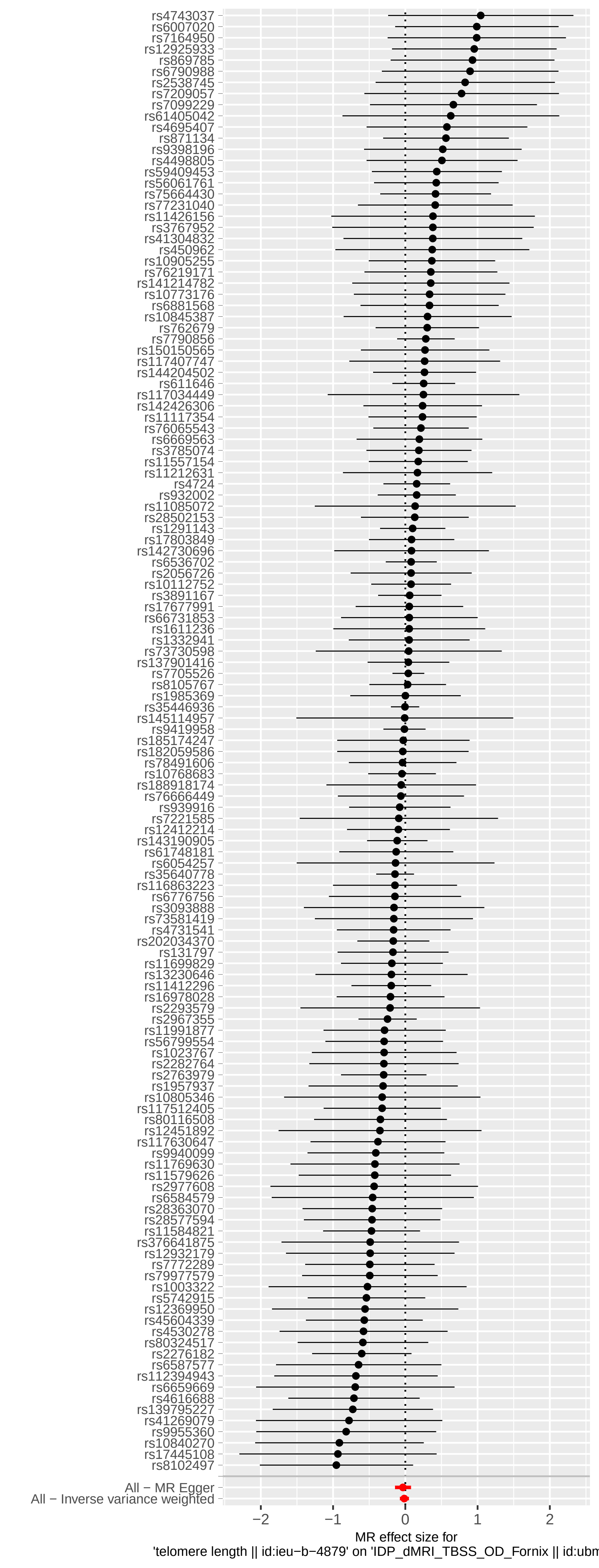

rs4743037
rs6007020
rs7164950
rs12925933
rs869785
rs6790988
rs2538745
rs7209057
rs7099229
rs61405042
rs4695407
rs871134
rs9398196
rs4498805
rs59409453
rs56061761
rs75664430
rs77231040
rs11426156
rs3767952
rs41304832
rs450962
rs10905255
rs76219171
rs141214782
rs10773176
rs6881568
rs10845387
rs762679
rs7790856
rs150150565
rs117407747
rs144204502
rs611646
rs117034449
rs142426306
rs11117354
rs76065543
rs6669563
rs3785074
rs11557154
rs11212631
rs4724
rs932002
rs11085072
rs28502153
rs1291143
rs17803849
rs142730696
rs6536702
rs2056726
rs10112752
rs3891167
rs17677991
rs66731853
rs1611236
rs1332941
rs73730598
rs137901416
rs7705526
rs8105767
rs1985369
rs35446936
rs145114957
rs9419958
rs185174247
rs182059586
rs78491606
rs10768683
rs188918174
rs76666449
rs939916
rs7221585
rs12412214
rs143190905
rs61748181
rs6054257
rs35640778
rs116863223
rs6776756
rs3093888
rs73581419
rs4731541
rs202034370
rs131797
rs11699829
rs13230646
rs11412296
rs16978028
rs2293579
rs2967355
rs11991877
rs56799554
rs1023767
rs2282764
rs2763979
rs1957937
rs10805346
rs117512405
rs80116508
rs12451892
rs117630647
rs9940099
rs11769630
rs11579626
rs2977608
rs6584579
rs28363070
rs28577594
rs11584821
rs376641875
rs12932179
rs7772289
rs79977579
rs1003322
rs5742915
rs12369950
rs45604339
rs4530278
rs80324517
rs2276182
rs6587577
rs112394943
rs6659669
rs4616688
rs139795227
rs41269079
rs9955360
rs10840270
rs17445108
rs8102497
All − MR Egger
All − Inverse variance weighted
−2
−1
0
1
2
MR effect size for
'telomere length || id:ieu−b−4879' on 'IDP_dMRI_TBSS_OD_Fornix || id:ubm
